# Divergent Forms of Pyroplastic: Lessons Learned from the M/V *X-Press Pearl* Ship Fire

**DOI:** 10.1021/acsenvironau.2c00020

**Published:** 2022-07-29

**Authors:** Bryan D. James, Asha de Vos, Lihini I. Aluwihare, Sarah Youngs, Collin P. Ward, Robert K. Nelson, Anna P. M. Michel, Mark E. Hahn, Christopher M. Reddy

**Affiliations:** † Department of Marine Chemistry and Geochemistry, Woods Hole Oceanographic Institution, Woods Hole, Massachusetts 02543, United States; ‡ Department of Biology, Woods Hole Oceanographic Institution, Woods Hole, Massachusetts 02543, United States; § Oceanswell, 9 Park Gardens, Colombo 5 00500, Sri Lanka; ∥ The Oceans Institute, University of Western Australia, 35 Stirling Highway, Perth, WA 6009, Australia; ⊥ Scripps Institution of Oceanography, University of California San Diego, La Jolla, California 92093, United States; # Department of Applied Ocean Physics and Engineering, Woods Hole Oceanographic Institution, Woods Hole, Massachusetts 02543, United States

**Keywords:** microplastic, resin pellets, pollution, additives, open burning, weathering, maritime accident

## Abstract

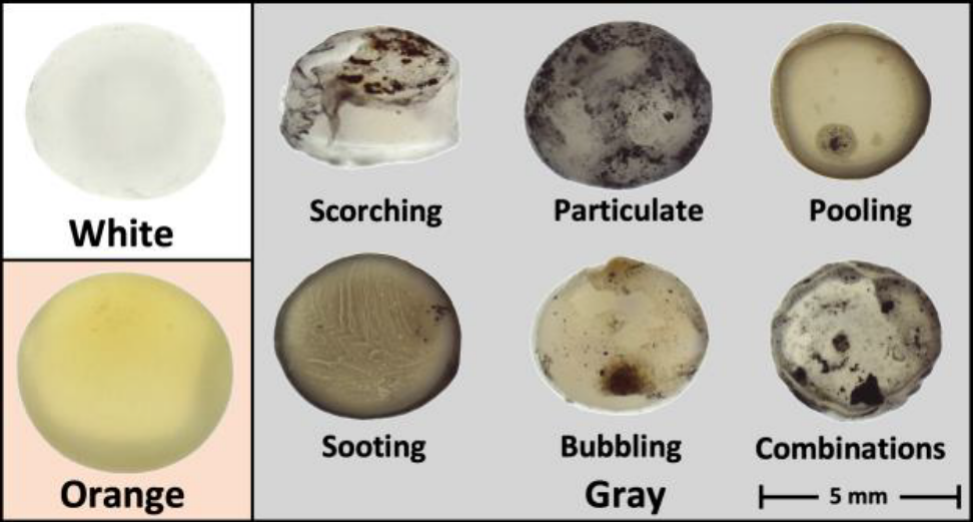

In late May 2021, the M/V *X-Press Pearl* container ship caught fire while anchored 18 km off the coast of Colombo, Sri Lanka and spilled upward of 70 billion pieces of plastic or “nurdles” (∼1680 tons), littering the country’s coastline. Exposure to combustion, heat, chemicals, and petroleum products led to an apparent continuum of changes from no obvious effects to pieces consistent with previous reports of melted and burned plastic (pyroplastic) found on beaches. At the middle of this continuum, nurdles were discolored but appeared to retain their prefire morphology, resembling nurdles that had been weathered in the environment. We performed a detailed investigation of the physical and surface properties of discolored nurdles collected on a beach 5 days after the ship caught fire and within 24 h of their arrival onshore. The color was the most striking trait of the plastic: white for nurdles with minimal alteration from the accident, orange for nurdles containing antioxidant degradation products formed by exposure to heat, and gray for partially combusted nurdles. Our color analyses indicate that this fraction of the plastic released from the ship was not a continuum but instead diverged into distinct groups. Fire left the gray nurdles scorched, with entrained particles and pools of melted plastic, and covered in soot, representing partial pyroplastics, a new subtype of pyroplastic. Cross sections showed that the heat- and fire-induced changes were superficial, leaving the surfaces more hydrophilic but the interior relatively untouched. These results provide timely and actionable information to responders to reevaluate cleanup end points, monitor the recurrence of these spilled nurdles, gauge short- and long-term effects of the spilled nurdles to the local ecosystem, and manage the recovery of the spill. These findings underscore partially combusted plastic (pyroplastic) as a type of plastic pollution that has yet to be fully explored despite the frequency at which plastic is burned globally.

## Introduction

In late May 2021, the M/V *X-Press Pearl* container ship caught fire off the coast of Colombo, Sri Lanka. When the fire started, the ship was carrying 1486 containers, of which 1214 held an assortment of raw materials, hazardous chemicals, and finished products.^1^ Included among the raw materials were 69 containers of polyethylene preproduction pellets or “nurdles” (32 containers of high-density grades and 37 containers of low-density grades), among other plastics. Five days after the fire started, ∼70 billion nurdles (∼1680 tons) and burnt plastic spilled from the ship and littered the Sri Lankan coastline.^1^ During the spill, the plastic was exposed to combustion, heat, chemicals, and petroleum products that led to what was initially described as a “burnt nurdle continuum” of debris.^1^ At the extremes of the continuum, the burnt plastic resembled pyroplastic, seemingly burned or melted brittle plastic of neutral color and geogenic appearance,^2^ or the plastic appeared untouched by the events of the fire. Pyroplastics have only recently been documented on beaches due largely to their camouflaged appearance and likely not to their rarity in the environment.^2−6^

Our initial assessment of the burnt plastic^1^ showed that it was polyethylene, was smaller and larger than nurdles, and was at least 3-fold more chemically complex than seemingly unburnt nurdles. Notably, we identified polycyclic aromatic hydrocarbons (PAHs) and petroleum-derived biomarkers and tentatively identified plastic additive degradation products in the burnt material. Phthalates and benzotriazole UV-stabilizers were not detected. We made several recommendations for the ongoing response based on our initial characterization of the seemingly unburnt nurdles and burnt plastic, including “[to] encourage locally led research efforts on nurdle samples to assess their physical and chemical properties, fate, and transport in the ocean and toxicity to aquatic life.”^1^

Following our initial findings, the International Pollutants Elimination Network (IPEN) in collaboration with the Sri Lankan Centre for Environmental Justice (CEJ) conducted additional chemical analyses on the nurdles and burnt plastic from four locations, detecting and quantifying trace metals (e.g., Fe, Ti, Cu), parent PAHs, a benzotriazole UV-stabilizer, and bisphenols.^7^ Residues from fluorinated fire-fighting foams were not detected. The report found different amounts of the detected species for different sample types and locations, which suggests that the material was much more heterogeneous than originally thought, thereby warranting refinement of how this plastic is described and organized for analysis.

While removal of the wreck is underway (estimated to be completed in 2023) and the cleanup has progressed, concerns persist about the hazardousness of the plastic.^8,9^ First person accounts underscore the concern for and confusion of those whose lives have been directly impacted by the spill, e.g., fishers and beachgoers.^7^ Nonetheless, nurdles in the ocean are not new.^10,11^ Past studies of oceanic nurdles have related their organic pollutant content and bioavailability, ingestion by wildlife, and degradative state to their color, suggesting that discrimination by color could resolve some of these outstanding worries.^12−15^ Concerns for the plastic’s hazardousness initially drove us to examine the nurdles discussed by de Vos et al.^1^ and motivated our continued in-depth analysis of them to offer further insight on the transformations undergone by the plastic. Clearly, the circumstances of the M/V *X-Press Pearl* maritime accident were harsher than those of earlier nurdle spills,^16−20^ which led to the release of a more complex pollutant.^1^ Having a robust characterization is necessary for any hazardous waste determination of the nurdles collected from beaches so that they are correctly sorted and classified.

Despite the tragedy of the spill, it provides a source of plastic of known origin that can be used to determine how exposure to heat and fire affects the environmental fate of plastic. Herein, our visual and surface chemical analyses of differentially colored nurdles collected within 24 h of arriving onshore during the first days of the ship fire reveal not a continuum of plastic, which would indicate a single transformation process, but several discrete forms of plastic suggestive of multiple separate processes. These divergent forms of pyroplastic would likely differ in their fate, persistence, and toxicity.

## Materials and Methods

### Materials

Nurdles were collected on Pamunugama Beach, Sri Lanka on May 25th, 2021 and shipped to the Woods Hole Oceanographic Institution.^1^ Prior to any measurements, nurdles were rinsed with Milli-Q (IQ 7000; Millipore Sigma) water and analytical grade ethanol to remove any debris and dried in a fume hood overnight. Nurdles were otherwise stored at 4 °C.

### Optical Microscopy

To quantify their color and morphological features, nurdles were first coarsely sorted by eye against a white background into groups based on their color. Then individual nurdles were illuminated on a tracing board and imaged using a relatively inexpensive 5-megapixel digital microscope (model 44308, Celestron). Each nurdle was imaged once from the random orientation that it took when set on the tracing board. Images were processed using the National Institutes of Health ImageJ (1.53f51) software, first by applying a default threshold to define the pixels associated with the nurdles, then using the particle analysis plugin to measure morphometrics (projected area, projected perimeter, and circularity). Concurrently, the plugin generated outlines for regions of interest (ROIs) defining the nurdles. The ROI outlines were then applied to unaltered versions of the same images of the nurdles. These images were converted to a gray scale image stack separating the image into its hue, saturation, and brightness (HSB) components with gray scale values ranging from 0 to 255. To quantify the color of the nurdles, the mean and standard deviation gray value for each HSB component was calculated only within the ROI for each image of each nurdle.

### Densitometry

The density of nurdles was measured by adapting the density-titration method.^21−23^ Briefly, Milli-Q water and analytical grade ethanol were mixed at 10 different mass fractions of ethanol to prepare standard solutions of ethanol and water with different densities. The density of each solution was measured by massing a known volume of solution on an analytical balance. The density of the solution was then calculated. This process was repeated for each solution to yield a standard curve (quadratic fit, *R*^2^ = 0.98) of mass fraction of ethanol versus solution density. A quadratic fit gave the best fit, as expected.^24^ Data points for the standard curve were measured in triplicate. To measure the density of each nurdle, a nurdle was placed in a known mass of Milli-Q water and ethanol was added dropwise with vigorous mixing between drops until the nurdle was neutrally buoyant. At this point, the mass of the added ethanol was measured, the mass fraction of ethanol in the solution was calculated, and using the previously prepared standard curve the density of the solution corresponding to the density of the nurdle was determined. The precision and accuracy of the method were ±0.001 g/cm^3^ (*n* = 3) and 0.4% (*n* = 1), respectively.

### Attenuated Total Reflectance-Fourier Transform Infrared Spectroscopy

Attenuated total reflectance-Fourier transform infrared spectroscopy (ATR-FTIR) was performed to assess chemical changes in the nurdles. One spectrum was collected for each nurdle. Spectra were recorded using a Bruker Invenio S FTIR instrument with a diamond crystal ATR module and acquired as the average of 64 measurements for 400–4000 cm^–1^ at a resolution of 4 cm^–1^. Spectra were postprocessed in OPUS 8.2.28 vibrational spectroscopy software using a standard extended ATR correction and baseline correction. To calculate spectral features (Table 1), spectra were exported from OPUS, numerically integrated in GraphPad Prism 9.3.1, and calculated in Microsoft Excel.

**Table 1:** Spectral Features Calculated for ATR-FTIR Spectra of Nurdles^a^

spectral feature	formula	ref
bond index R–OH	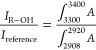	(25)
bond index C–O	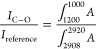	(25)
bond index C=C	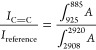	(25, 26)
branching index	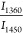	(27)
crystallinity (%)	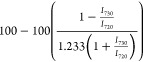	(28, 29)
polyethylene type	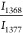	(30)

a I = intensity; A = absorbance.

### Contact Angle Goniometry

Water contact angle goniometry of sessile droplets was used to assess the relative hydrophilicity of nurdles. An approximately 1 μL droplet of Milli-Q water was manually placed on the nurdles. Droplet volume and positioning were optimized for each nurdle to minimize the impact of nurdle curvature, which could lead to the droplet moving along the surface. Images of the droplets were taken using a Biolin Scientific Attension Theta Lite Optical Tensiometer, and contact angles were calculated using the OneAttension software. The precision and accuracy of the method were ±1.685° (*n* = 5) and 4.7 ± 1.7% (*n* = 5), respectively.

### Statistical Analyses

Statistical analyses were conducted using GraphPad Prism 9.3.1. When appropriate, either parametric or nonparametric tests were used to compare groups. Groups were considered significantly different for a *p* value less than 0.05. Sample sizes and statistical tests are included in the text and figure captions where appropriate.

## Results and Discussion

Nurdles collected 5 days after the M/V *X-Press Pearl* caught fire and within 24 h of arrival onshore were analyzed by image-based colorimetry and morphometry, densitometry, infrared spectroscopy, and contact angle goniometry.

### Color

In the field and laboratory,^13,31−34^ several strategies have been used to categorize nurdles with various levels of detail from coarse color groups (e.g., red, blue, yellow) to finer interpolated color palettes (e.g., white, tan, light yellow, dark yellow). As is common practice by International Pellet Watch and others,^33,35^ we first sorted the nurdles into broad groups based on color, either being white, orange, or gray. Within each group there was noticeable variation observable by eye. It has been shown that using finer palettes and indices (e.g., yellowness or whiteness) to further resolve color can correlate with a plastic’s exposure to degradative processes.^32−34,36^

**Figure 1:**
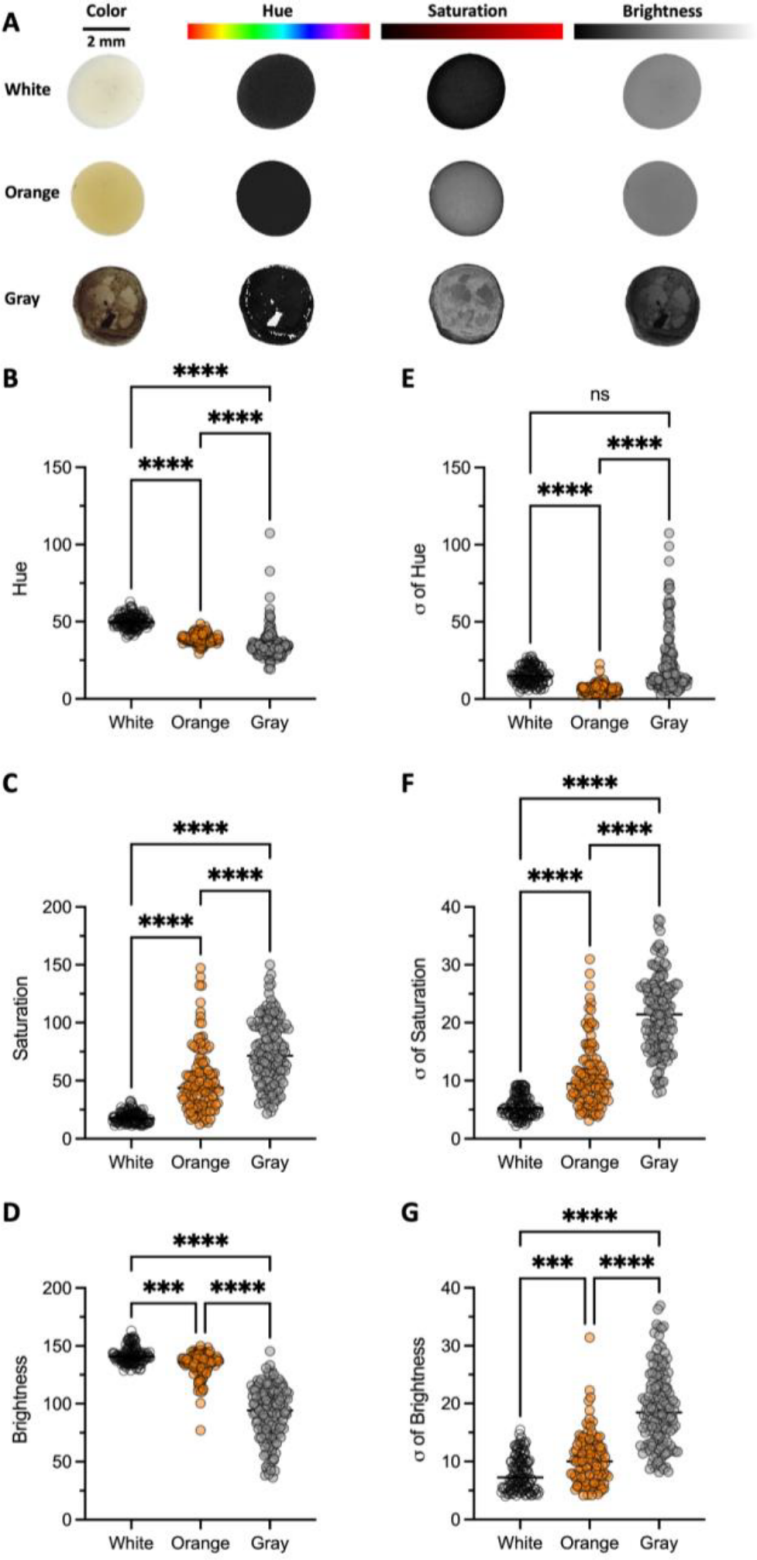
Quantification of color. Representative color images (A) of white, orange, and gray nurdles and their color separated into components of hue, saturation, and brightness. Distributions of the mean hue (B), saturation (C), and brightness (D) for nurdles from each color group. Each component was statistically different for the three groups. Distributions of the variability of hue (E), saturation (F), and brightness (G) across each nurdle’s surface from the three groups was reported as the standard deviation (σ). For white nurdles, *n* = 135; for orange nurdles, *n* = 115; for gray nurdles, *n* = 149. Comparisons were made using a Kruskal–Wallis test with Dunn’s test for multiple comparisons. *** = *p* < 0.001, **** = *p* < 0.0001, ns = not significant.

Building on this, we employed a method applied in the biomedical sciences for studying cells and reimagined it for interrogating nurdles. We have argued that such interdisciplinary exchanges can be exceedingly fruitful for studying plastics in the environment.^37^ As would be done for stained or fluorescently labeled cells,^38,39^ we deconstructed a nurdle’s color into its hue, saturation, and brightness (HSB) components. Rarely has this been done for images of plastic particles and, even if so, using all three components.^40^ Using this quantitative approach, we resolved a nurdle’s color with hyperfine precision (i.e., 256^3^ unique HSB combinations). Moreover, rather than compare a nurdle’s average color to a color palette,^41^ we used image-based quantification from high-resolution microscope images to resolve color in terms of HSB at every pixel associated with the nurdle in the image (Figure 1A). This yielded a simple and repeatable method for discriminating nurdle color that could quantify not only gross color, but its heterogeneity across a nurdle’s surface offering a more rigorous description of a nurdle’s color. This approach is not exclusive to nurdles and has the potential to be repeated by those investigating other forms of microplastics.

Color among nurdles in each group was similar in hue and varied more in saturation and brightness (Figures 1B–D, S1A–C). Orange and gray nurdles were more variable within their color groups. The standard deviation of each nurdle’s hue, saturation, and brightness was used as a measure of color heterogeneity across the nurdle’s surface. White and gray nurdles were the most homogeneous and heterogeneous, respectively (Figures 1E–G, S1D,F). Orange nurdles were homogeneous in hue and heterogeneous in saturation and brightness. In the context of a color-producing compound, hue is related to the absorbance spectrum, saturation is related to the width of the absorbance band, and brightness is related to the absorbance intensity.^42^ Homogeneity in hue and relatively low heterogeneity in saturation suggests that the color-producing compounds were the same within and among the nurdles. Heterogeneity in brightness suggests that the amount of the color-producing compounds varied in distribution within the nurdles. Gray nurdles varied the most, emphasizing that color was heterogeneous for a given nurdle and within the color group.

### Morphology

**Figure 2:**
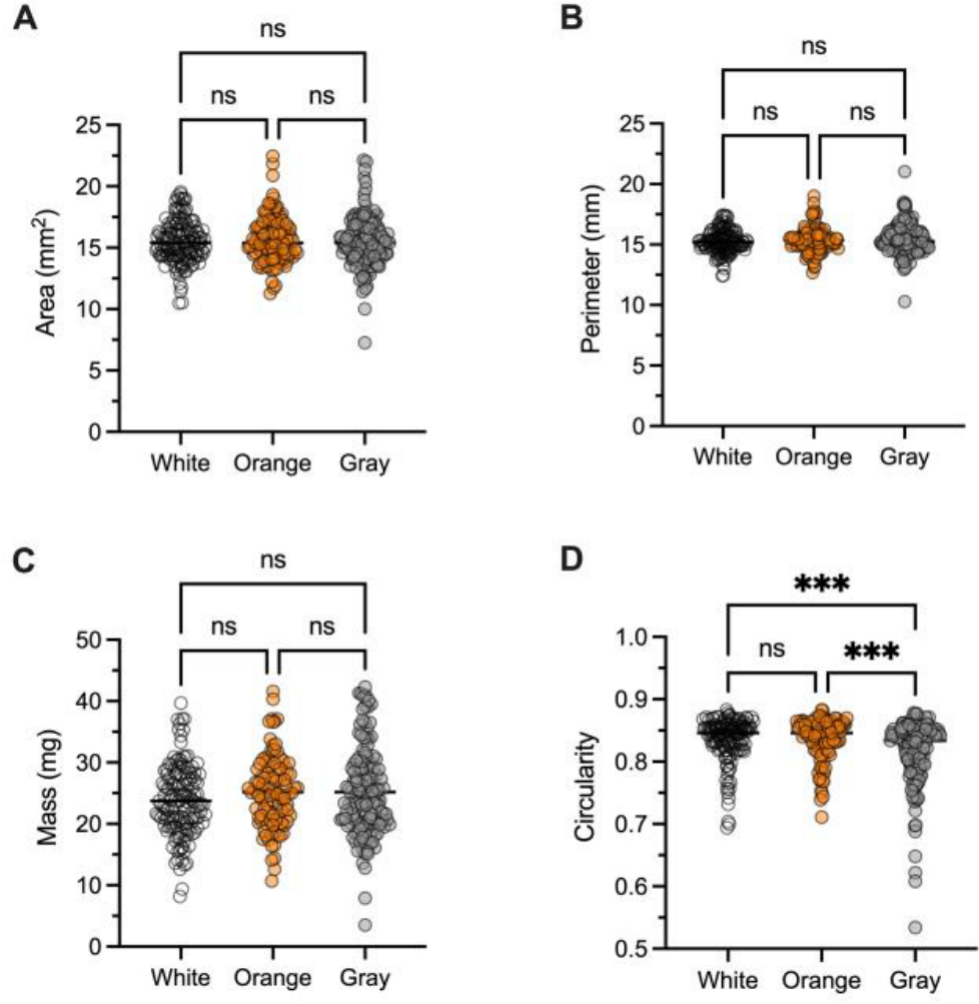
Shape and size. There was no significant difference in nurdle projected area (A), projected perimeter (B), and mass (C) for the color groups. Gray nurdles were slightly less circular (D) than orange and white nurdles (*p* < 0.001). For white nurdles, *n* = 135 (morphometrics) and *n* = 140 (mass); for orange nurdles, *n* = 115 (morphometrics) and *n* = 108 (mass); for gray nurdles, *n* = 149 (morphometrics) and *n* = 140 (mass). Comparisons were made using a Kruskal–Wallis test with Dunn’s test for multiple comparisons. *** = *p* < 0.001, ns = not significant.

Despite the variety of color, there was little to no difference in the size and shape of the nurdles among the three color groups (Figure 2). Nurdles ranged in projected area from 10.48 to 22.47 mm^2^, projected perimeter from 10.28 to 21.03 mm, and mass from 8.2 to 42.3 mg. Gray nurdles were less circular (Mdn: 0.8340, IQR: 0.7958–0.8513) than white nurdles (Mdn: 0.8460, IQR: 0.8260–0.8580) and orange nurdles (Mdn: 0.8846, IQR: 0.8240–0.8600), suggesting that their shape had been deformed by the fire. There was no correlation between the shape and size of the nurdles and HSB (Figures S2, S3).

### Distribution of Pyroplastics Released during the M/V *X-Press Pearl* Spill

Having a baseline distribution of each color group is important for monitoring efforts to account for the plastic from the spill. The distribution of each color group in a random ∼44 g (∼1760 equiv-nurdles assuming 25 mg per nurdle) subsample of spilled plastic was ∼83.8% white nurdles, ∼0.9% orange nurdles, ∼4.5% gray nurdles, and ∼10.7% burnt plastic (Figure S4). If this distribution is assumed to be representative of the total population of plastic that was spilled, then this corresponds to 1408 tons of white nurdles, 15 tons of orange nurdles, 76 tons of gray nurdles, and 180 tons of burnt plastic that were spilled. Thus, both the orange and gray nurdles should not be discounted because of their small fraction (<5%), because each group would be considered a sizable nurdle spill on its own. For comparison, previous nurdle spills from container ships have ranged from 13 tons (Norway, 2020)^20^ to 150 tons (Hong Kong, 2012).^19^

### Nurdle Taxonomy

**Figure 3:**
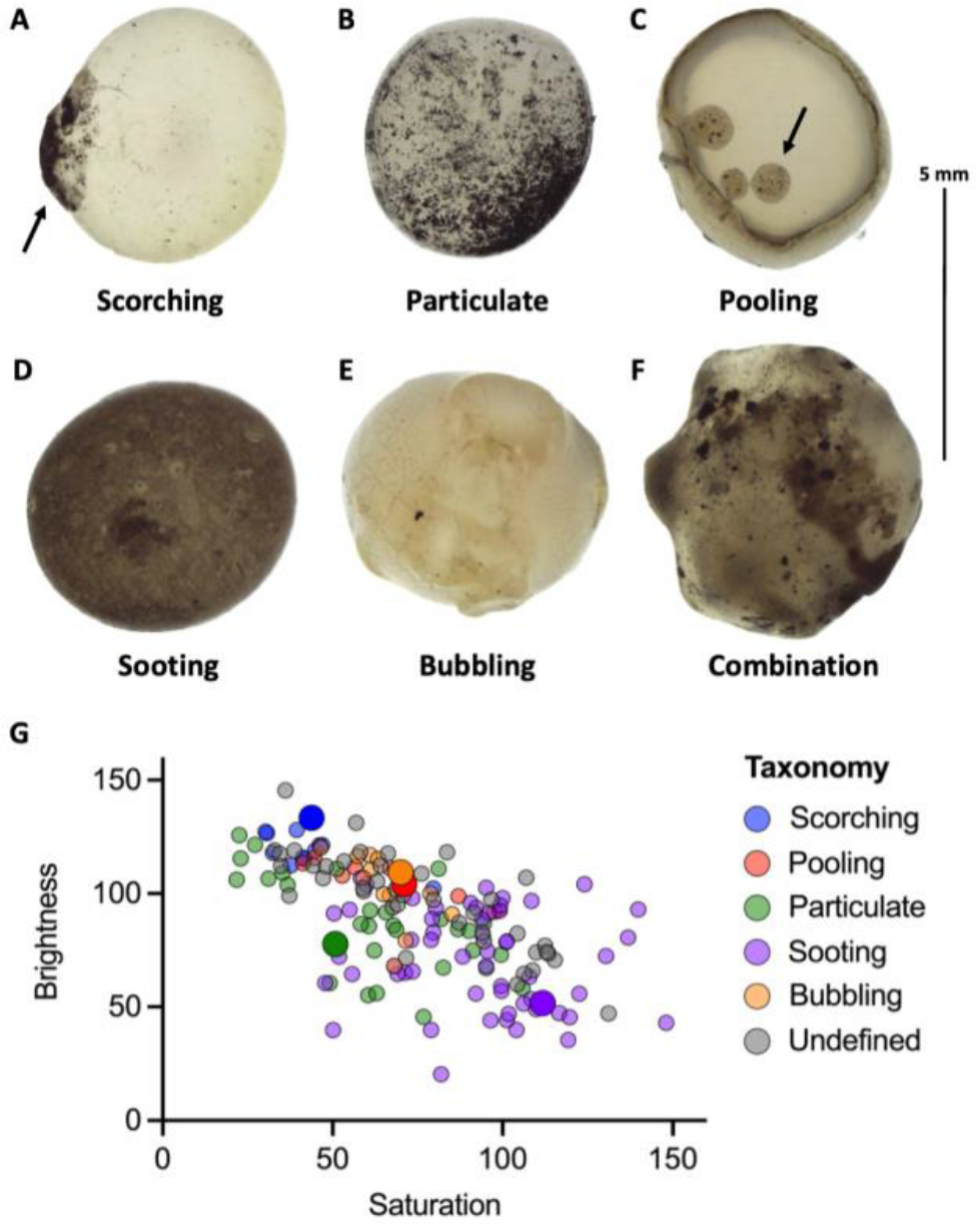
Taxonomic groups. Gray nurdles could show scorching (A), entrained particulate (B), pools of melted plastic (C), sooting (D), bubbling (E), and combinations of any of these features (F). Plot (G) of saturation versus brightness for gray nurdles shown in Figure 1 color coded to their taxonomic group. Larger circles correspond to the nurdles shown in A-E. Those labeled “undefined” were nurdles that did not fit any of the taxonomic groups. Complete HSB quantification for the taxonomic groups is presented in Figure S5.

The heterogeneity in the HSB of the gray nurdles appeared to be related to their many different surface features (Figure 3). We observed many recurring features and so defined a taxonomy that included *scorching* in which a localized region of the nurdle experienced partial combustion (Figure 3A); *entrained particulate* in which distinct small particles are embedded on or in the nurdle (Figure 3B); *pooling* in which the surface of the nurdle has small, localized splotches of particulate and discolored plastic (Figure 3C); *sooting* in which the majority of the surface is covered in a fine, black soot (Figure 3D); or *bubbling* in which the surface of the nurdle is distorted and cracked (Figure 3E); and combinations of them (Figure 3F). HSB values of representative nurdles with these features were distinct, except for those of pooling and bubbling, which overlapped (Figure 3G). Despite such differences, there was no clear relationship between HSB and these taxonomic groups when extended to all gray nurdles (Figure S5). This was attributed to nurdles being combinations or partial combinations like that in Figure 3F (Supporting Information). Recognizing the specific features of the gray nurdles should help to identify pyroplastics more broadly among microplastics.

### Physical and Chemical Properties

A random subset of nurdles from each color group was analyzed for their density, infrared (IR) spectrum, and water contact angle to determine whether these properties varied with color.

#### Density

**Figure 4:**
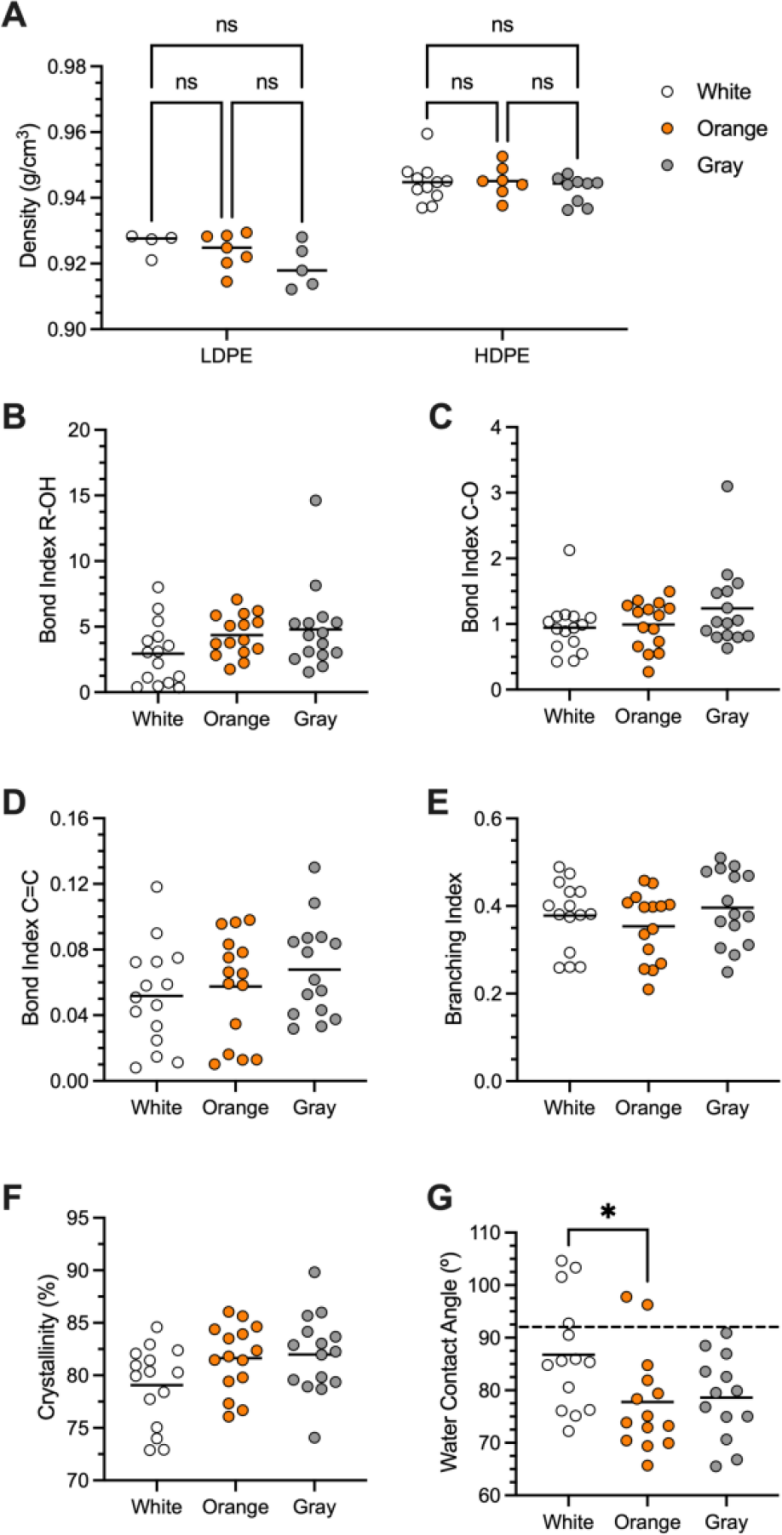
Density (A). There was no difference in density between the color groups for each type of polymer (LDPE and HDPE). LDPE was defined as density less than 0.93 g cm^–3^, and HDPE was defined as density greater than 0.93 g cm^–3^. There was a statistical difference (*p* < 0.0001) in density between polymer type. Comparisons were made using a two-way ANOVA with Šídák test for multiple comparisons. For white nurdles, *n* = 15; for orange nurdles, *n* = 14; for gray nurdles, *n* = 14. ns = not significant. LDPE = low-density polyethylene. HDPE = high-density polyethylene. IR features and indices (B–F). The bond index for R–OH (B), the bond index for C–O (C), the bond index for C=C (D), the branching index (E), and crystallinity (F) were calculated using the formulas presented in Table 1. Comparisons were made using a one-way Brown–Forsythe and Welch’s ANOVA with Dunnett’s T3 test for multiple comparisons. For white nurdles, *n* = 15; for orange nurdles, *n* = 15; for gray nurdles, *n* = 15. Water contact angle (G). The dotted line corresponds to that of reference virgin LDPE (Goodfellow).^51,52^ Comparison made using a one-way ANOVA with Tukey test for multiple comparisons. For white nurdles, *n* = 14; for orange nurdles, *n* = 14; for gray nurdles, *n* = 13. * = *p* < 0.05.

The density of the nurdles separated into two classes, those that were (1) less than ∼0.93 g/cm^3^ and (2) greater than ∼0.93 g/cm^3^. These values reflect the density of low-density polyethylene (LDPE) and high-density polyethylene (HDPE), respectively.^43^ Within these two classes, nurdles spanned the gamut of color, yet there was no difference in density among the color groups (Figure 4A). Similarly, there was no correlation between density and HSB (Figures S2, S3). It should be noted that we do not know the original density of these nurdles; however, product sheets for the Lotrène brand of polyethylene nurdles that were listed on the ship manifest^1^ provide some clues that the densities for LDPE and low linear-density polyethylene (LLDPE) nurdles could range from 0.918 to 0.923 g/cm^3^ and HDPE nurdles could range from 0.946 to 0.964 g/cm^3^ (Supporting Information).

#### IR Spectroscopy

ATR-FTIR is often used for polymer identification and assessing weathering indices for thermal degradation and surface-level crystallinity of plastic. The IR spectra for 15 random nurdles from each color group were indicative of polyethylene (Figure S6), and by using the ratio of the integrated peak area at 1368 cm^–1^ to the integrated peak area at 1377 cm^–1^ we determined that nurdles were either HDPE or LDPE, assuming that the conditions of the fire did not alter the relative abundance of those signals. For most of the nurdles, this spectroscopic analysis corroborated our assignment of polyethylene type for the nurdles as determined by density (Figure S7).

Peaks associated with weathering were apparent. Nurdles from each color group included peaks for hydroxyls from broad medium peaks at 3350 cm^–1^ (R–OH) and 1100 cm^–1^ (C–O) belonging to a mixture of primary, secondary, and tertiary alcohols. Peaks at 1640 and 910 cm^–1^ were indicative of vinyl groups. Interestingly, there were few defined peaks between 1650 and 1750 cm^–1^, suggesting little accumulation of carbonyls (C=O).^44^ The absence of carbonyl groups is not surprising. Nonstabilized, thermally aged HDPE has been reported to accumulate few carbonyl groups in extruders at 260 °C while accumulating vinyl groups.^45^ Such processing conditions likely mirror the conditions the nurdles may have experienced during the ship fire, including elevated temperature and low oxygen content.^46^ Additionally, hydroperoxide degrading antioxidants commonly used in polyethylene can also contribute to this effect.^47^ These findings corroborate our previous assessments of solvent extracts from white nurdles measured by comprehensive two-dimensional gas chromatography,^1^ which showed enrichment of alkenes, not typically found in extracts from new polyethylene products, rather than alkanes.^48^

Established features and indices were calculated for the spectra to quantify the extent of any chemical changes. Indices for hydroxyl (R–OH), carbon–oxygen (C–O), and vinyl (C=C) groups, branching, and crystallinity were calculated (Table 1). A carbonyl peak was not detectable and thus a carbonyl index was not calculated. Interestingly, there were no statistical differences among the color groups for any of the indices, although some trends existed (Figure 4). We attributed the lack of statistical significance among groups to the large degree of scatter in the measurements. The variability was likely due to surface heterogeneities (Figure 3) as well as spatial limitations of our ATR-FTIR instrument, which warrants pursuing IR spatial mapping to resolve in greater detail the surface functionalities of the nurdles. Separating by polymer type (low-density vs high-density) did not change this result, except in the case of crystallinity (Figure S8). Gray nurdles were more crystalline than white nurdles when separated by type (*p* < 0.01) (Figure S8E). Crystallinity had a strong negative correlation with hue (Figures S2, S3). This further reveals that image-accessible information (e.g., color, morphology) is a rich source of information that correlates with and characterizes microplastic physical and chemical properties.^49^ Importantly, indices for R–OH and C–O had a strong positive correlation with one another, consistent with hydroxyl formation. Similarly, the correlation between indices for C=C and short chain branching was consistent with thermal degradation of the polymer (Figures S2, S3). Under thermal degradative conditions (240–60 °C), processes that form vinyl groups and branches both occur.^45^ Collectively, the spectra demonstrate that the surface level chemical changes were not unique to one grade of polyethylene or color group.

#### Hydrophilicity

Functionally, the presence of hydroxyls on the surface of the degraded nurdles was predicted to make them more hydrophilic. To test this, we measured water contact angle (Figure 4G). Contact angle measurements are dependent on the properties of the surface (physical and chemical),^50^ the large scatter in the data suggests heterogeneity of the nurdle surfaces. There was no statistical difference in water contact angle between LDPE and HDPE. There was a statistical difference between white and orange nurdles (*p* = 0.0441) and nearly one between white and gray nurdles (*p* = 0.0791), while there was no difference between orange and gray nurdles (*p* = 0.9740). The mean water contact angle for all color groups was less than that of reference LDPE, suggesting that the nurdles were more hydrophilic. These results complement the IR measurements substantiating the introduction of hydrophilic hydroxyl groups to the surface. Overall, these data support the idea that the degraded nurdles are variable within their color group and that visible color does not expressly indicate the extent of degradation; even white nurdles could be appreciably degraded on their surface due to the heat of the fire.

### Origin of the Orange Color: Heat and Antioxidants

The yellowing and pinking of nurdles found on beaches globally is often attributed to the formation of quinoidal transformation products from phenolic antioxidants.^36,53,54^ These additives help inhibit polymer degradation by hydroperoxides and other radicals that form during processing at elevated temperatures and weathering in the environment.^47,53−56^ In nature, photochemical weathering is the dominant process for degrading polyolefins.^44^ However, samples were collected within days of the spill (a timeline much shorter than rates of natural photochemical weathering),^52^ implying that these color changes are likely not the result of photochemical degradation and more likely resulted from thermal degradation.

The presence of hydroxyls and absence of carbonyls in the IR spectra of the nurdles suggests that an antioxidant was active because antioxidants degrade carbonyl forming species (hydroperoxides) to yield hydroxyls.^47^ Additionally, nurdle aggregates were observed (Figure S9), supporting the notion that the nurdles experienced heat sufficient to melt and sinter together during the spill. At least one polyethylene grade listed onboard the ship (Lotrène FD0274) is known to contain antioxidants among other additives, including a slip additive (∼600 ppm erucamide) and antiblocking additives (∼900 ppm) (Supporting Information).

In our preliminary assessment of solvent extracts from white “unburnt” nurdles by comprehensive two-dimensional gas chromatography,^1^ we tentatively determined the presence of 2,4-di-*tert*-butylphenol, a degradation product of tris(2,4-di-*tert*-butylphenyl)phosphite (Trade names: Irgafos 168 or Hostanox PAR 24), a ubiquitous phosphite antioxidant.^57,58^ This suggested that the phosphite was likely the antioxidant used in the spilled plastic. Unlike in the International Pollutants Elimination Network (IPEN) report,^7^ we did not detect any benzotriazole UV-stabilizers, affirming the significant variability of the spilled plastic.

As tris(2,4-di-*tert*-butylphenyl)phosphite scavenges hydroperoxides in the polymer, the molecule oxidizes to its phosphate form and the polymer backbone accumulates a hydroxyl group.^47,59^ Thus, because of the fire, the nurdles, regardless of discoloration, were expected to contain hydroxyls and be more hydrophilic than virgin polyethylene. Both tris(2,4-di-*tert*-butylphenyl)phosphite and tris(2,4-di-*tert*-butylphenyl)phosphate, the oxidized form, are susceptible to hydrolysis by conditions likely present during the ship fire that would yield 2,4-di-*tert*-butylphenol.^47,58−63^ While the phosphate and phosphite do not lead to discoloration and may even lead to whitening,^63^ 2,4-di-*tert*-butylphenol can form discoloring quinoidal compounds,^47,64−66^ the likely source of the orange color in the nurdles. Thus, we conclude that the heat from the fire and the antioxidants contained within the nurdles together led to the orange color.

### Origin of the Gray Color: Fire and Partial Combustion

The mechanisms that gave the orange color do not explain the gray discoloration, pointing to the fire as its source. Unlike in other reports of pyroplastics,^2^ which focused on the complete melting and burning of plastic, we propose that the variety of features observed for the gray nurdles stem from short, direct contact or proximity to fire. Burning of polymers is characterized by a melt zone with pyrolytic conditions below the flame with adjacent char regions.^67−70^ These processes are the probable cause of the scorching, particulate, and bubbling features. Pooling could be from localized melting due to a nearby flame or from drips of melted, burnt plastic falling on them. Dispersed among the plastic collected from the beach were tiny spherical droplets of plastic (Figure S10), which could have dripped from burning polyethylene. Soot is generated in scenarios of incomplete combustion due to low oxygen content. Ship fires are characterized by poor ventilation, making the production of soot inevitable.^46^ The fire aboard the *X-Press Pearl* was no exception, generating thick plumes of dark smoke,^71^ which were the probable sources of the soot found on some of the gray nurdles. We conclude that partial combustion of the nurdles led to their gray color.

### Superficial Transformations

For polyethylene, density is related to its crystallinity (i.e., LDPE is less crystalline than HDPE).^43^ During thermal degradation, the amorphous region of the polymer is attacked preferentially, so we expected to observe an increase in the crystallinity of the polymer as well as the density for the more degraded nurdles (orange and gray).^72,73^ This was not apparent from our bulk density measurements (Figure 4A). However, the degradation may have only been superficial (changing the surface of the polymer and not the bulk of the polymer) and therefore may not have been substantial enough to affect the bulk density of the degraded nurdles.

**Figure 5:**
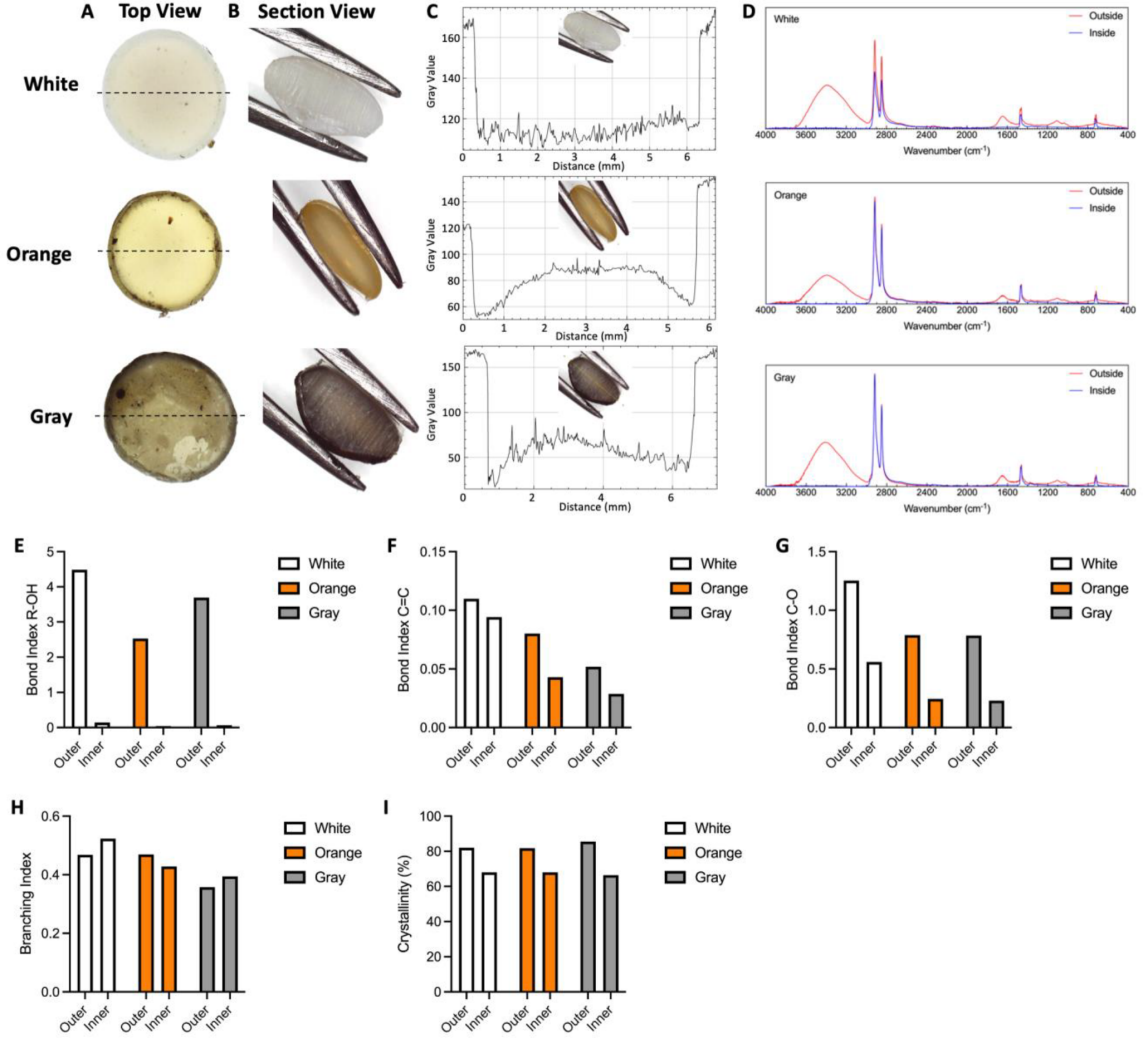
Cross sectional analysis. Top view images of the representative nurdles from each color group (A). Dashed lines are the approximate sectioning line. Section view images of the nurdles with the interior face shown (B). Line scans of pixel intensity highlight that the nurdle discoloration was mostly restricted to within the first millimeter of thickness (C). IR absorption spectra for the outside and inside surfaces of the sections (D). Quantified spectral features and indices for the outside and inside surfaces of the nurdles (E–I). One nurdle was examined from each color group.

To determine whether these changes were exclusive to the surface of the nurdles, representative nurdles from each color group were sectioned in half and imaged and their interior surface was assessed by IR spectroscopy. Images of the interior revealed that the discoloration was largely superficial (within 1 mm of the surface) (Figure 5A,B). Line scans of the color intensity across the thickness of the interior surface showed that, for the orange and gray nurdles, there was less discoloration at the center as compared to the edges (Figure 5C). Comparison of IR spectra from the exterior and interior surfaces revealed that the nurdles were not degraded through their entire thickness (Figure 5D) and indices reflected this difference (Figure 5E–I). This result is interesting because thermal degradation would conceivably occur throughout the polymer matrix, but heat and mass transfer processes during the fire could have limited temperature and reactants (O_2_). Additionally, thermal oxidation is a heterogeneous process that works inward, and these plastics contained antioxidants, both of which could have contributed to the preservation of the internal polymer.^74,75^ Unfortunately, we have no record of the exact degradation conditions and can only assess the gross outcome of the ship fire.

### Implications for the M/V *X-Press Pearl* Accident

Prior to the spill, nurdles were rarely found on Sri Lankan beaches.^76−81^ The tremendous influx of differentially degraded plastic pellets raises questions about the impact on wildlife and the fate of the plastic in the environment.

The color of a nurdle can dictate its interaction with wildlife. Animals that perceive color from below could be more prone to ingest darker nurdles while those that perceive color from above could be more prone to ingest lighter nurdles.^82^ Though discrepancies exist for this generalization and may prove to be species-specific, it underscores that color can be a significant determinant for ingestion by wildlife.^12,83^ In contrast, filter feeding organisms such as bivalves, corals, and anemones show less consideration for color and ingest and egest plastic based more on the physical properties (shape and size) and surface properties (weathering and biofilm maturity) of the plastic.^84−87^ Because these nurdles are similarly shaped and sized, surface properties may be a driving factor of their consumption by filter feeders. Likewise, because these nurdles have similar densities, shapes, and sizes, it is likely they will travel in the ocean in the same way regardless of their color.^1^

Nurdle color is associated with the amount of harmful pollutants that accumulate on nurdles in the ocean.^13,34,36^ So far, nurdles from the *X-Press Pearl* spill have been shown to contain at least PAHs, a UV-stabilizer (2-*tert*-butyl-6-(5-chloro-2-benzotriazolyl)-4-methylphenol; trade names: bumetrizole, tinuvin 326), bisphenol A (4,4′-(propane-2,2-diyl)diphenol), and several metals.^1,7^ Importantly, each of these is known to negatively affect health.^88−91^ The amount of these species, particularly the PAHs, was dependent on the collection location. For the 12 parent PAHs measured by IPEN, nurdles collected north of the wreck had ∼800 ng/g compared to ∼50 ng/g for nurdles collected south of the wreck.^7^ These amounts of PAHs are within the range of those measured by the International Pellet Watch for nurdles found globally.^92^ The source of the PAHs was not determined, though, given the nature of the maritime accident, both petrogenic and pyrogenic sources are possible. A more definitive assessment requires measurement of at least parent PAHs and their alkylated homologues.^93^ Despite one report that heavily weathered nurdles (dark orange in color) have reduced bioavailability of organic pollutants,^34^ the bioavailability of these chemicals, others, and those yet to be identified in the spilled *X-Press Pearl* nurdles remains unknown. Thus, the color changes and the organic pollutants already detected in the nurdles further necessitate evaluating the toxicity of this plastic.

Hazard assessments of the spilled plastic should consider nurdle color. Nurdles and other plastic particles are sinks for organic pollutants (e.g., PAHs and polychlorinated biphenyls) in the ocean and because of this are routinely used to monitor their presence.^33,92,94^ Interactions between plastic and organic pollutants are dependent on the surface properties of the plastic;^34,95−97^ thus, having a more hydrophilic surface may affect this process. Past work has correlated nurdle color to its loading of organic pollutants;^13,34,36^ however, correlation of nurdle color with organic pollutant content will likely not be possible in the short term as nurdles from this spill have entered the environment already discolored from pretreatment by heat and fire. Specifically, the convention used by the International Pellet Watch for measuring organic pollutants in nurdles is to analyze pollutants only in pellets with a yellowness index greater than 40.^33^ This cutoff is used to ensure that the nurdles have been in the environment for an amount of time sufficient to allow accumulation of pollutants (as indicated by their orange/yellow color arising presumably from photochemical degradation). If this same approach was applied to the nurdles from this spill, it may lead to discrepancies because shorter term thermal alterations could be mistaken for longer term photochemical changes. It is unclear how these past approaches to nurdle pollution apply to color changes derived from thermal treatment, a key research priority moving forward.

Treatment by heat and fire changed the surface of the nurdles before they spilled into the environment, leading to uncertainties in their environmental fate. Because only the surface has been degraded, it is expected that these nurdles will behave differently, at least for a period. Once erosive processes strip the nurdles of their degraded layer, revealing undegraded plastic, the nurdles will likely behave as their virgin counterparts do in the marine environment by gradually yellowing and cracking on their surface.^98^ The duration of this period and the future stability of these nurdles is also likely altered because some of their phosphite antioxidant has been consumed and hydrolyzed to yield the partially hindered phenolic antioxidant, 2,4-di-*tert*-butylphenol, which can act to mitigate oxidation of the polymer and lead to further discoloration. In contrast, being more colored may hasten photochemical degradation because visible wavelengths of light can be absorbed from newly acquired chromophores, as has been demonstrated for polystyrene.^99^ Thus, the future rates and pathways of photochemical degradation may differ for each color group.

Another potential consequence of the heat- and fire-altered surface of the nurdles is enhanced fragmentation to smaller microplastic and nanoplastic particles, as previously suggested for pyroplastic.^2^ It is well-known that fragmentation can be accelerated by weathering processes such as exposure to UV irradiation and mechanical abrasion.^100−102^ Susceptibility of plastics to mechanical fragmentation can be enhanced by exposure to high temperatures.^103^ Smaller particles are more likely to be taken up by organisms, increasing the potential for toxicity.^104,105^ Moreover, the physical and chemical properties of pyroplastic-derived particles are likely to differ from those generated from unburned plastic, altering their toxicological properties. For example, the high concentrations of PAHs present on some pyroplastic-derived nanoplastics could lead to enhanced toxicity.^106^ Understanding the toxicological implications of pyroplastic fragmentation is an important topic in need of further research.

Heat and fire likely changed the plastic’s microbiological interactions and capacity for microbial degradation, too. Thermal degradation results in chain scission and oxygenation of polyethylene, which likely increased the lability of the surface of the plastic to microbial attack. In fact, Indian-Ocean-derived species of bacteria have been shown to degrade thermally treated LDPE and HDPE.^107^ Similarly, the increased hydrophilicity from oxygen-containing functional groups will likely affect the eco-corona and biofilm that would typically form on virgin nurdles in the ocean.^108,109^ As environmental processes act on the plastic, the physical and chemical variation of the nurdles may continue to diverge or reconverge, necessitating that analyses moving forward assess samples from different locations and time points following the spill to monitor the fate of this plastic.

Nurdles will continue to change color from photochemical weathering and this point should be recognized so that nurdles are rightfully associated with this spill. Being superficial, the gray fraction of plastic may, in time, appear to go away while the orange fraction may, in time, appear to increase from photochemical degradation of the nurdles. Future monitoring efforts should consider this important nuance when assessing the impacts of the spill.^15^ A first estimate of the proportion of plastic types based on their color and appearance (white nurdles, orange nurdles, gray nurdles, and burnt plastic) revealed that a sizable mass of orange and gray nurdles may have been released. Assessing the proportion of white, orange, and gray nurdles, and burnt plastic across time and location may provide insight about the fate of the different fractions in the environment.

Overall, many properties of the degraded nurdles were independent of color. The nurdles had variable surface features and surface chemistries, and they were different from virgin plastic, reinforcing the idea that these spilled pellets are different than those from other spills and sources.^1,16−20^ Importantly, fire and elevated temperature created several features (scorching, entrained particulate, pools of melted plastic, sooting, and bubbling) on the surface of the nurdles without altering their bulk physical properties, presenting as pieces of partial pyroplastic, a new subtype of pyroplastics.

### Implications Globally

Thermally altered (orange), partially combusted (gray), and burnt plastic can enter the marine environment not only from a maritime accident like that of the M/V *X-Press Pearl* but from more frequent open burning of waste and forest fires. Only recently has pyroplastic been reported on beaches likely because of its camouflaged appearance rather than its absence.^2−5^ Such a material likely arises from the open burning of an estimated 970 million tons of plastic-containing waste each year.^110,111^ Similarly, natural disasters, especially wildfires, can be another source of pyroplastic entering the environment.^112−114^ Forms of plastic like those from the *X-Press Pearl* are likely produced from these actions, and remnants of this material can then make their way into the environment along the same paths as other mismanaged waste.^115^ The environmental uncertainties concerning the spilled plastic from the *X-Press Pearl* accident also apply to divergent forms of pyroplastics globally. Plastics were already considered diverse contaminants^116^ and are made more diverse from weathering (e.g., heat, combustion, light) (Figure S11). The incomplete combustion and melting of plastic yields material with a broad range of surface features distinct from those of photoweathered plastic and with uncertain environmental impacts that have yet to be appreciably explored.

## Supporting Information Available

Distributions of the shape, size, and mass of nurdles, images of fused nurdles, distributions of the density of nurdles, matrices of Spearman *r* coefficients and their *p* values for correlations of color and measured properties of the nurdles, ATR-FTIR spectra of nurdles, distributions of the spectral indices for the nurdles, compilation of product sheets accessible on January 26, 2022 from the Qatar Petrochemical Company (QAPCO) and QChem suppliers of some of the plastic onboard the M/V *X-Press Pearl* (PDF)
