# Supplementary material for: Divergent Forms of Pyroplastic: Lessons Learned from the M/V X-Press Pearl Ship Fire
Source: ACS Environ Au. 2022 Jul 29;2(5):467–79. doi: 10.1021/acsenvironau.2c00020 (PMC10125272; doi:10.1021/acsenvironau.2c00020)
Supplement: Supplementary file 1 — vg2c00020_si_001.pdf [file vg2c00020_si_001.pdf]

Supporting Information

Divergent Forms of Pyroplastic: Lessons Learned from the M/V *X-Press Pearl* Ship Fire

Bryan D. James[a, b, *], Asha de Vos[c, d], Lihini I. Aluwihare[e], Sarah Youngs[f], Collin P. Ward[a], Robert K. Nelson[a], Anna P. M. Michel[f], Mark E. Hahn[b], Christopher M. Reddy[a]

[a] Department of Marine Chemistry and Geochemistry, Woods Hole Oceanographic Institution, Woods Hole, Massachusetts 02543, United States
[b] Department of Biology, Woods Hole Oceanographic Institution, Woods Hole, Massachusetts 02543, United
[c] Oceanswell, 9 Park Gardens, Colombo 5, Sri Lanka
[d] The Oceans Institute, University of Western Australia, 35 Stirling Highway, Perth, WA, 6009, Australia
[e] Scripps Institution of Oceanography, University of California San Diego, La Jolla, California, 92093, United States
[f] Department of Applied Ocean Physics and Engineering, Woods Hole Oceanographic Institution, Woods Hole, Massachusetts 02543, United States

* Corresponding author information:
Bryan D. James: bjames@whoi.edu

No. of pages: 12
No. of figures: 11

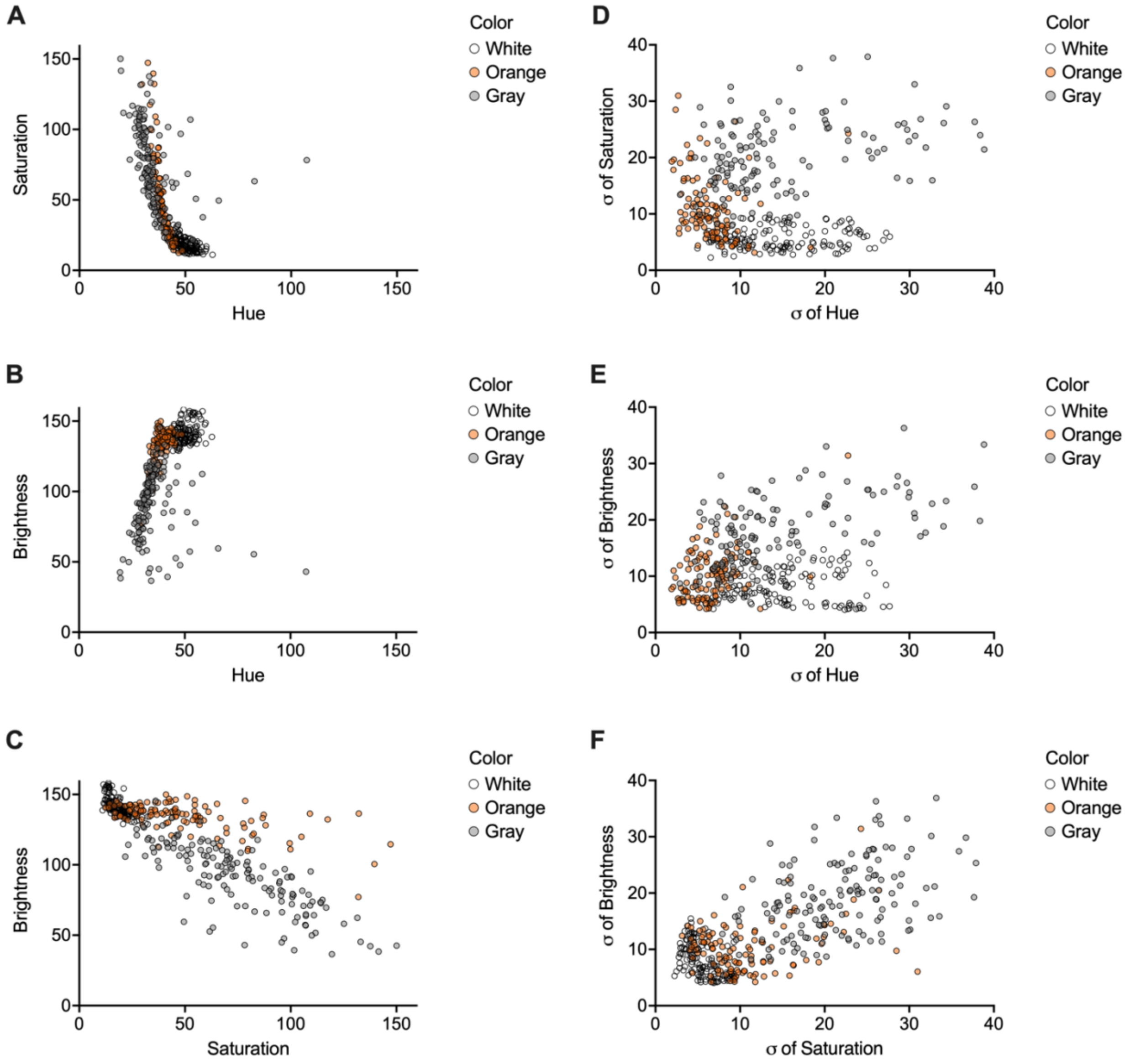

Figure S1. Pairwise plots of hue, saturation, and brightness (A-C). Pairwise plots of the standard deviation (σ) of hue, saturation brightness (D-F). For white nurdles, n = 135; for orange nurdles, n = 115; for gray nurdles, n = 149.

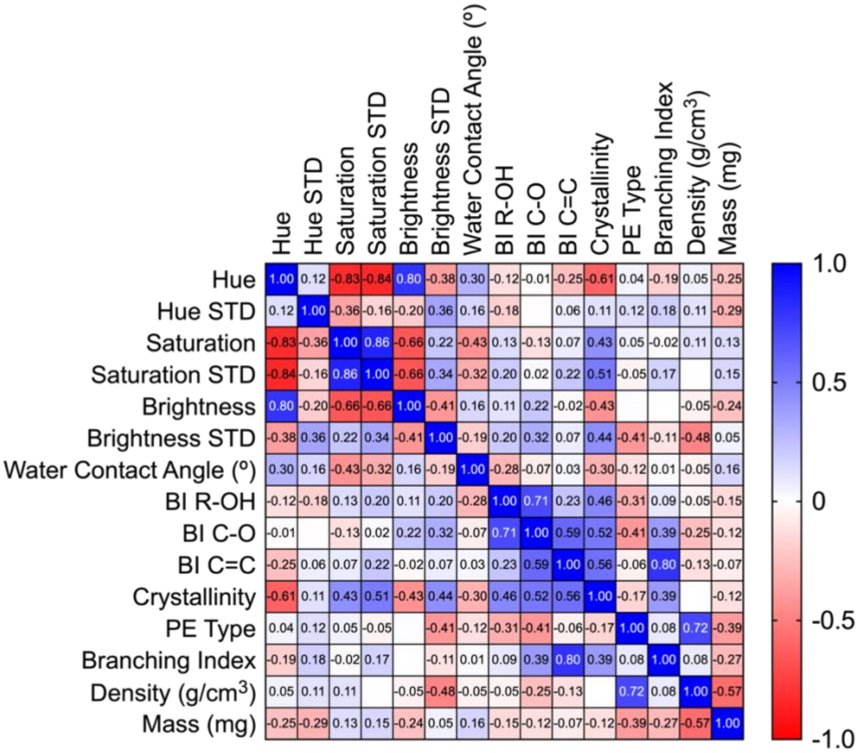

Figure S2. Matrix of Spearman *r* coefficients.

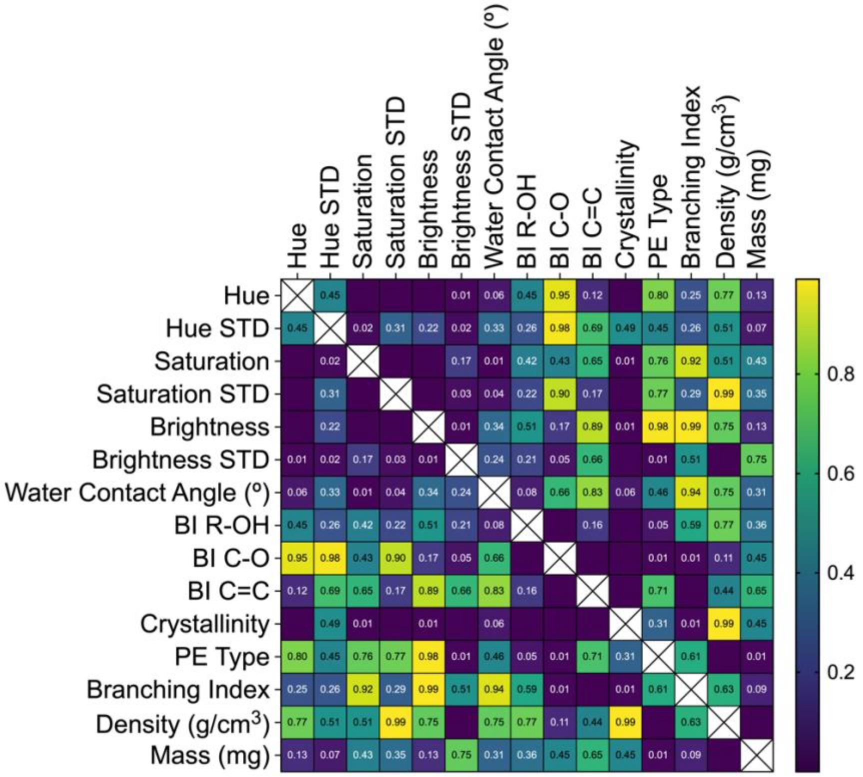

Figure S3. Matrix of *p* values for Spearman *r* coefficients ($p < 0.05$ indicates significance).

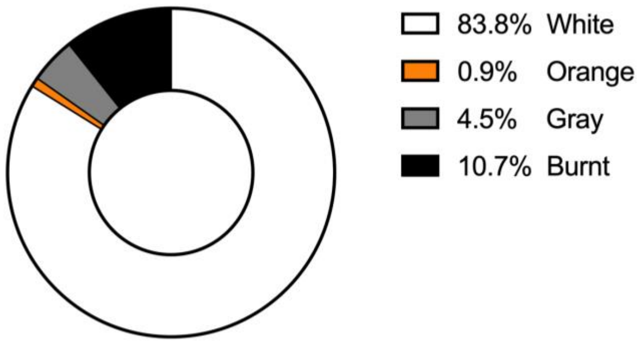

Figure S4. Pyroplastic distribution. Initial estimate for the proportion of white, orange, and gray nurdles and burnt plastic that spilled. This assessment was made from a random subsample of ~44 g of spilled plastic. Assuming an average mass of 25 mg per nurdle the approximate sample size of this mass of plastic (assuming that it was made of only nurdles) is equivalent to ~1760 nurdles. For the total population of nurdles spilled (~70 billion), this sample size gives a 96% confidence interval for these values with a 99% confidence level. Sample size was calculated from a Z-score for a 99% confidence interval using an assumed standard deviation of 0.5 to ensure representation of the population, $sample\ size\ = \frac{(Z-score)^2\sigma(1-\sigma)}{(1-confidence\ interval)^2}$

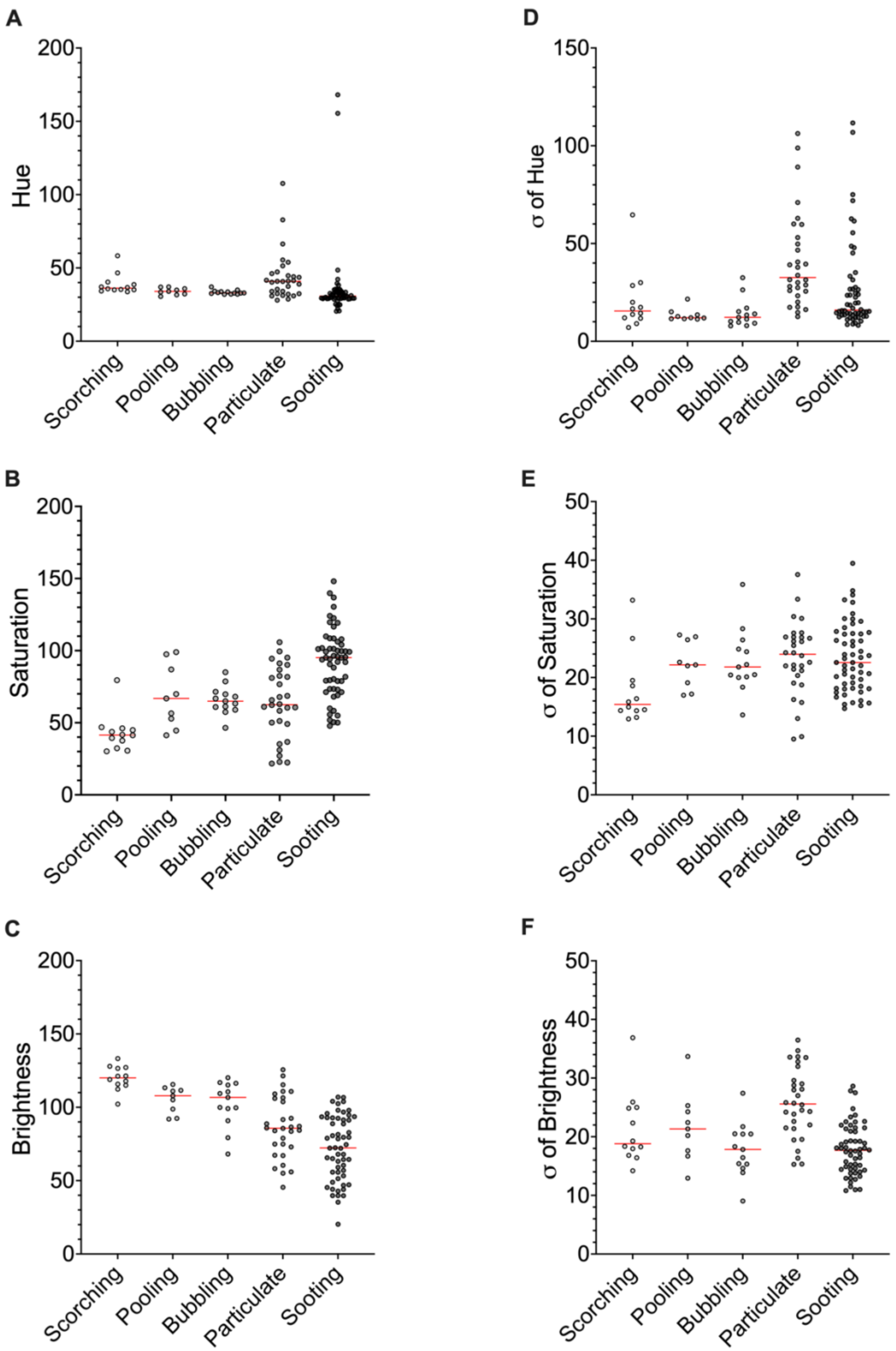

Figure S5. HSB quantification of gray nurdle taxonomic groups. Red lines indicate the median value.

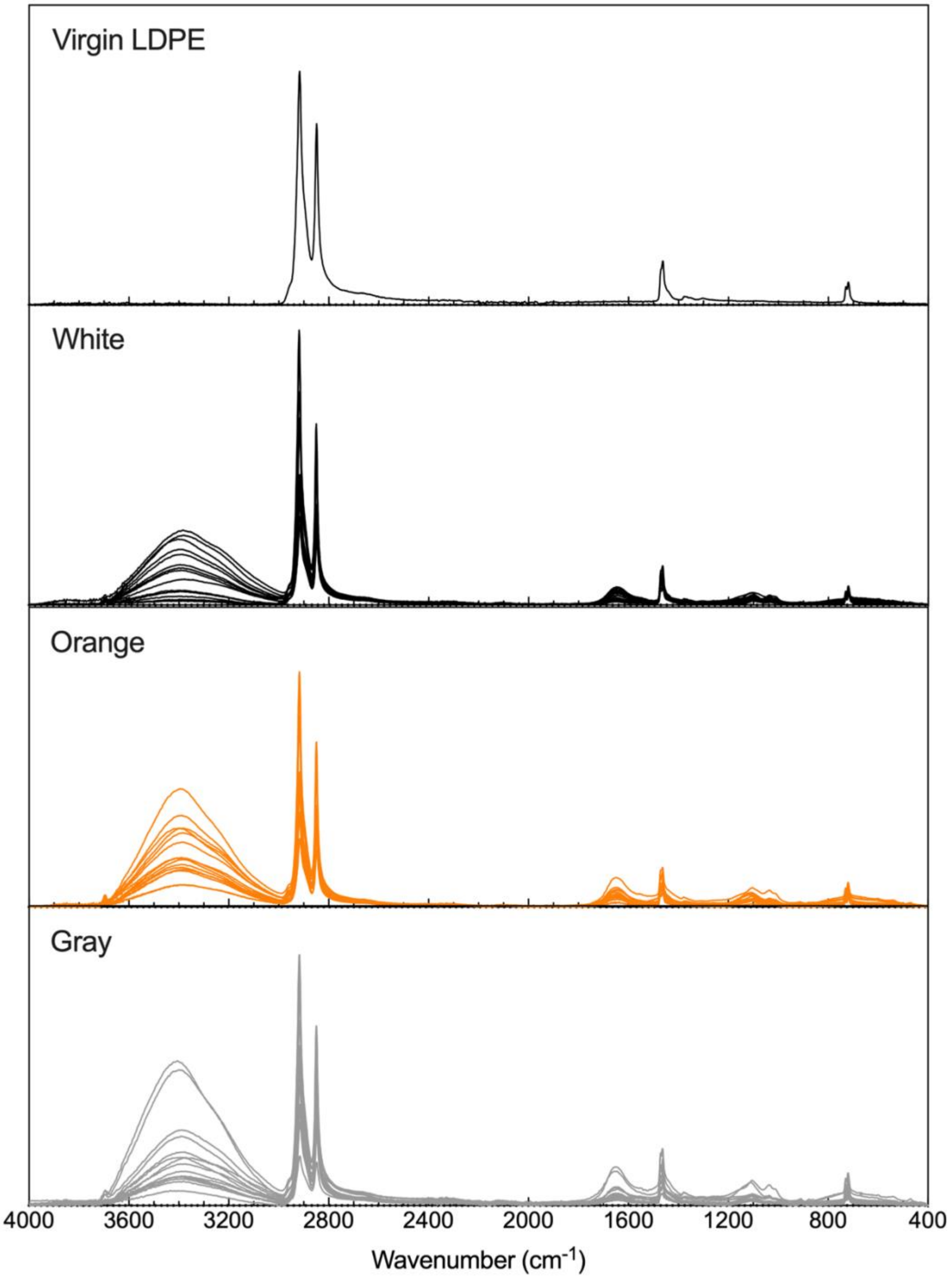

Figure S6. ATR-FTIR spectra of nurdles. Peaks at 3350 cm^{-1} correspond to R-OH bonds, at 1640 cm^{-1} and 910 cm^{-1} correspond to C=C bonds, and at 1100 cm^{-1} correspond to C-O bonds. All spectra are consistent with that of polyethylene. An additive-free LDPE film (Goodfellow) was used as a virgin LDPE reference.

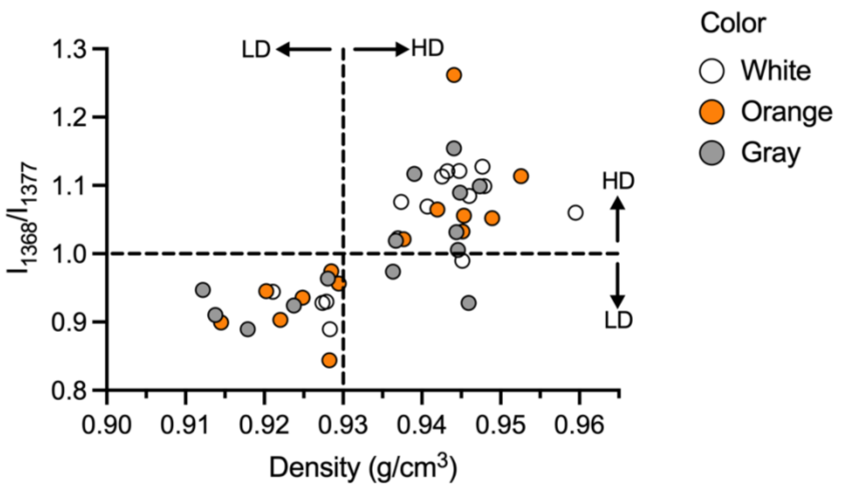

Figure S7. Comparison of polyethylene grade assignment based on density and on ratio of I_{1368} to I_{1377}. A density < 0.93 g/cm^3 was considered LDPE and > 0.93 g/cm^3 was considered HDPE. A ratio of I_{1368} to I_{1377} <1 was considered LDPE and >1 was considered HDPE.

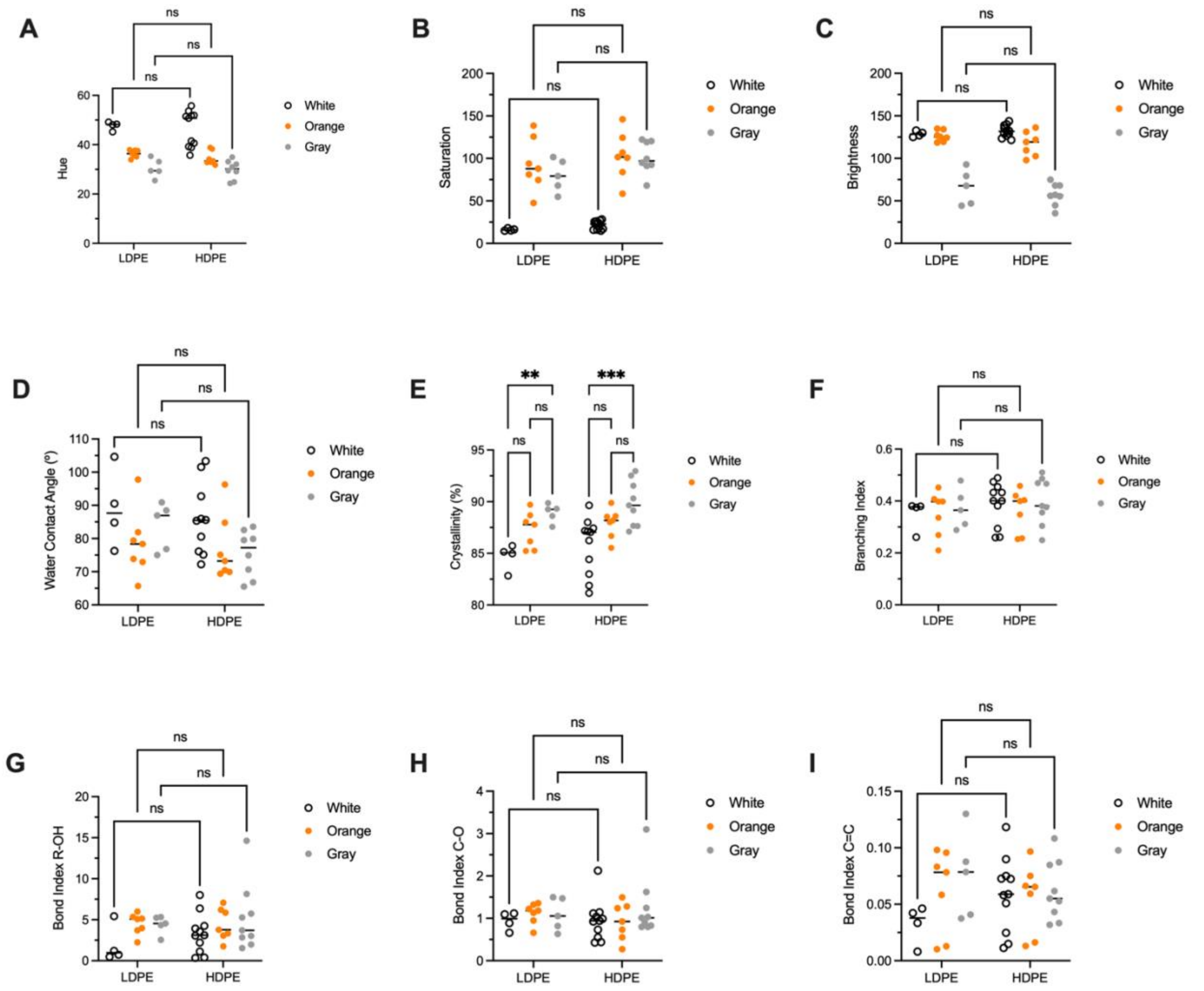

Figure S8. Differences in measured properties as a function of polyethylene type as determined by density. Features and indices were calculated using the formulas presented in **Table 1**. Comparisons were made using a two-way ANOVA with Šídák test for multiple comparisons. ** = p<0.01, *** = p<0.001, ns = not significant.

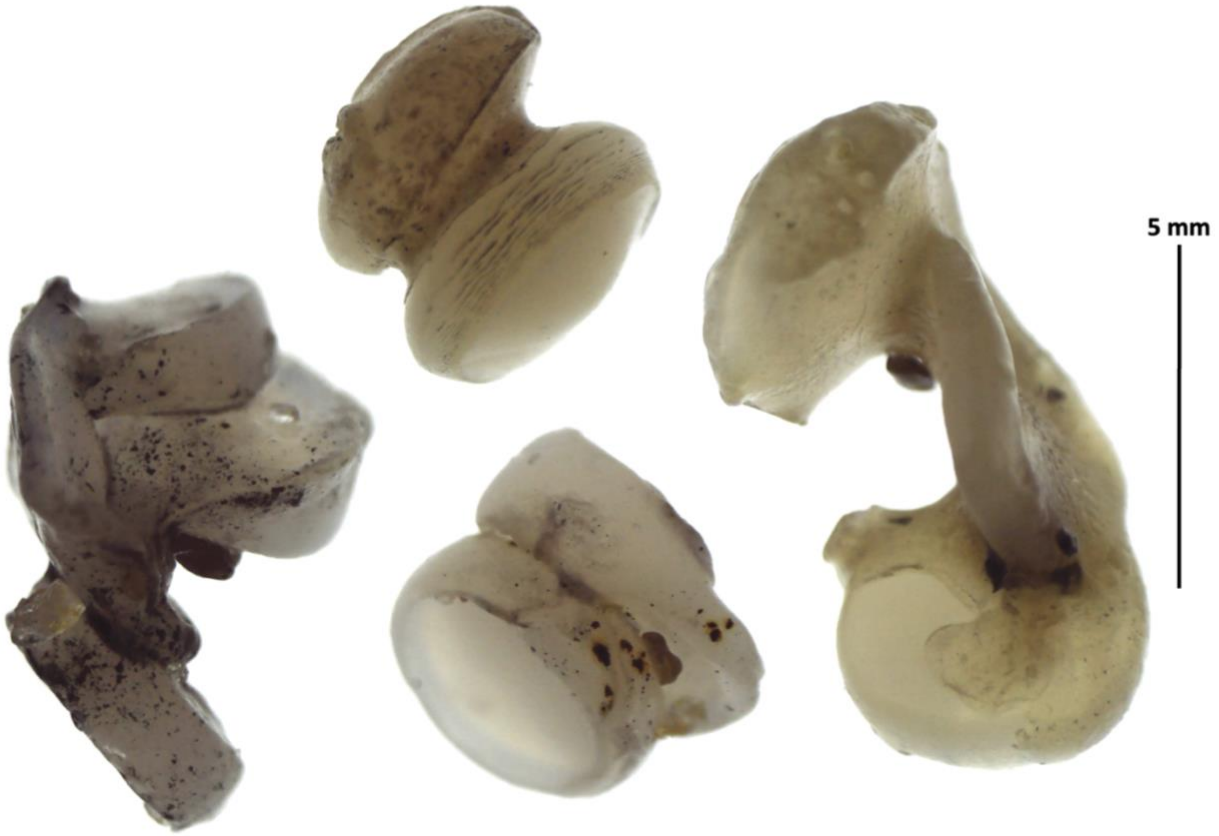

Figure S9. Images of fused nurdles. These nurdles display evidence of scorching (**Figure 3A**), entrained particulate (**Figure 3B**), sooting (**Figure 3D**), and bubbling (**Figure 3E**).

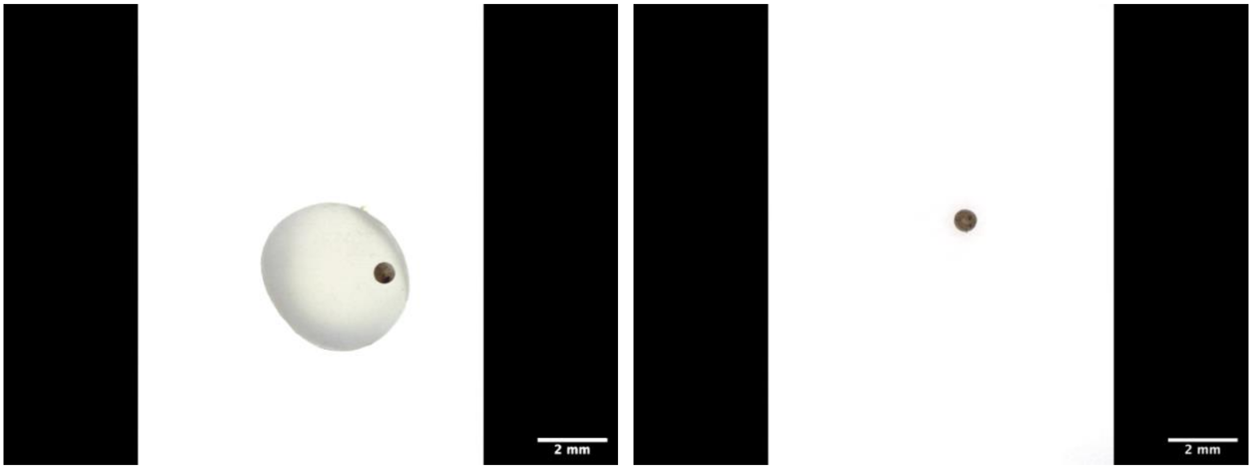

Figure S10. Droplets of melted, burnt plastic found among the nurdles.

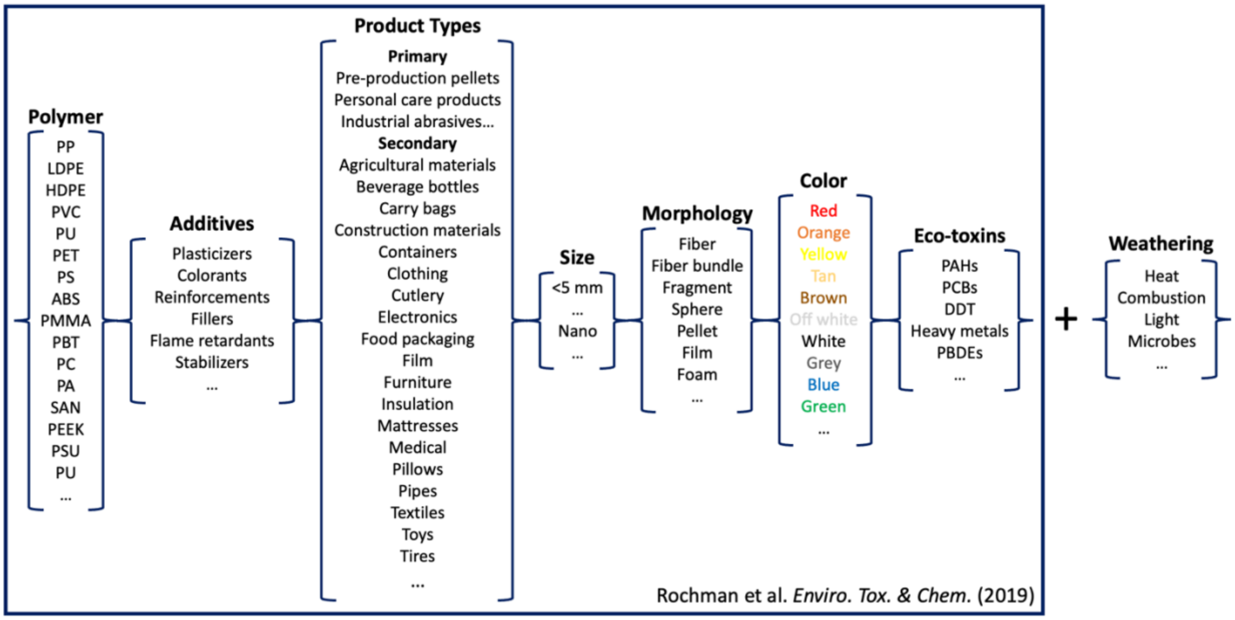

Figure S11. Microplastics are a diverse contaminant that is made more complex by weathering (e.g., heat, combustion, light, microbes, etc.). Adapted with permission from Chelsea M. Rochman, Cole Brookson, Jacqueline Bikker, et al. Rethinking microplastics as a diverse contaminant suite. *Environmental Toxicology and Chemistry*. 2019, 38, 4. Copyright 2019 John Wiley and Sons.

Potential plastic onboard the M/V *X-Press Pearl*

The ship manifest[1] provided details of potential resin pellet products or "nurdles" that were washing ashore on May 25, 2021 onto Pamunugama Beach, Sri Lanka. Specifically, this forensic evidence indicated that at least a portion of the nurdles were from the Lotrène brand of polyethylene sold by Qatar Petrochemical Company (QAPCO) and QChem. According to each company's website in total 12 grades of low-density polyethylene (LDPE), three grades of linear low-density polyethylene (LLDPE), and four grades of high-density polyethylene (HDPE) are sold under the brand name. Product sheets for each grade revealed that the nurdles could range in density from 0.918 to 0.923 g/cm^3 for LDPE[2–13] and LLDPE[14–16] and range from 0.946 to 0.964 g/cm^3 for HDPE.[17–20] As other properties and details may be useful for the response, we have compiled a list with URL links for the product sheets of the 19 grades of Lotrène branded polyethylene products advertised by QAPCO and QChem as of January 26, 2022. References 2-13 correspond to LDPE grades, references 14-16 correspond to LLDPE grades, and references 17-20 correspond to HDPE grades.

References

(1) de Vos, A.; Aluwihare, L.; Youngs, S.; DiBenedetto, M. H.; Ward, C. P.; Michel, A. P. M.; Colson, B. C.; Mazzotta, M. G.; Walsh, A. N.; Nelson, R. K.; Reddy, C. M.; James, B. D. The M/V X-Press Pearl Nurdle Spill: Contamination of Burnt Plastic and Unburnt Nurdles along Sri Lanka’s Beaches. *ACS Environmental Au* **2022**, *2* (2), 128–135. https://doi.org/10.1021/acsenvironau.1c00031.
(2) QAPCO. *Lotrène MG20 LDPE*. http://www.muntajat.qa/uploads/lotrene/Lotrène MG20 2014 04 16.pdf (accessed 2022-01-26).
(3) QAPCO. *Lotrène LA0710 LDPE*. https://qapco.com/storage/2020/07/Datasheet_LA0710-2.pdf (accessed 2022-01-26).
(4) QAPCO. *Lotrène FB3003 LDPE*. https://qapco.com/storage/2020/07/Datasheet_FB3003-2.pdf (accessed 2022-01-26).
(5) QAPCO. *Lotrène FB5026 LDPE*. https://qapco.com/storage/2020/07/Datasheet_FB5026-2.pdf (accessed 2022-01-26).
(6) QAPCO. *Lotrène FD0270 LDPE*. https://qapco.com/storage/2020/07/Datasheet_FD0270-2.pdf (accessed 2022-01-26).
(7) QAPCO. *Lotrène FD0274 LDPE*. https://qapco.com/storage/2020/07/Datasheet_FD0274-2.pdf (accessed 2022-01-26).
(8) QAPCO. *Lotrène FD0374 LDPE*. https://qapco.com/storage/2022/05/Datasheet_FD0374-3.pdf (accessed 2022-01-26).
(9) QAPCO. *Lotrène FD0474 LDPE*. https://qapco.com/storage/2020/07/Datasheet_FD0474-2.pdf (accessed 2022-01-26).
(10) QAPCO. *Lotrène FE3000 LDPE*. https://qapco.com/storage/2020/07/Datasheet_FE3000-2.pdf (accessed 2022-01-26).
(11) QAPCO. *Lotrène FE8000 LDPE*. https://qapco.com/storage/2020/07/Datasheet_FE8000-2.pdf (accessed 2022-01-26).
(12) QAPCO. *Lotrène MG70 LDPE*. https://qapco.com/storage/2022/05/Datasheet_MG70-4.pdf (accessed 2022-01-26).
(13) QAPCO. *Lotrène FE8004 LDPE*. https://qapco.com/storage/2020/07/Datasheet_FE8004-2.pdf (accessed 2022-01-26).
(14) QAPCO. *Lotrène Q1018 LLDPE*. https://qapco.com/storage/2020/07/Datasheet_Q1018-2.pdf (accessed 2022-01-26).
(15) QAPCO. *Lotrène Q2018 LLDPE*. https://qapco.com/storage/2020/07/Datasheet_Q2018-2.pdf (accessed 2022-01-26).
(16) QAPCO. *Lotrène Q2018C LLDPE*. https://qapco.com/storage/2020/07/Datasheet_Q2018C-2.pdf (accessed 2022-01-26).

(17) QChem. *Lotrène Q 50100 HDPE*. https://www.qchem.com.qa/App_Documents/Polyethylene/TDS/Q 50100.pdf (accessed 2022-01-26).
(18) QChem. *Lotrène Q TR-144 HDPE*. https://www.qchem.com.qa/App_Documents/Polyethylene/TDS/Q TR-144.pdf (accessed 2022-01-26).
(19) QChem. *Lotrène Q TR-571 HDPE*. https://www.qchem.com.qa/App_Documents/Polyethylene/TDS/Q TR 571.pdf (accessed 2022-01-26).
(20) QChem. *Lotrène Q 5502BN HDPE*. https://www.qchem.com.qa/App_Documents/Polyethylene/TDS/Q 5502 BN.pdf (accessed 2022-01-26).
